# The preclinical study of predicting radiosensitivity in human nasopharyngeal carcinoma xenografts by 18F-ML-10 animal- PET/CT imaging

**DOI:** 10.18632/oncotarget.7868

**Published:** 2016-03-03

**Authors:** Xiao Bao, Zhongyi Yang, Siyang Wang, Yujia Zheng, Mingwei Wang, Bingxin Gu, Jianping Zhang, Yongping Zhang, Yingjian Zhang

**Affiliations:** ^1^ Department of Nuclear Medicine, Fudan University Shanghai Cancer Center, Shanghai 200032, China; ^2^ Department of Oncology, Shanghai Medical College, Fudan University, Shanghai 200032, China; ^3^ Center for Biomedical Imaging, Fudan University, Shanghai 200032, China; ^4^ Shanghai Engineering Research Center for Molecular Imaging Probes, Shanghai 200032, China

**Keywords:** nasopharyngeal carcinoma, nude mouse, ^18^F-ML-10, animal-PET/CT, irradiation sensitivity

## Abstract

Previous studies have reported that the radiosensitivity is associated with apoptosis. Hereby, we aimed to investigate the value of ^18^F-ML-10 PET/CT, which selectively targeted cells undergoing apoptosis, in predicting radiosensitivity of human nasopharyngeal carcinoma (NPC) xenografts. We used CNE1 (highly differentiated) and CNE2 (poorly differentiated) NPC cell lines to construct tumor models, which had very different radiosensitivities. After irradiation, the volumes of CNE2 tumors decreased significantly while those of CNE1 tumors increased. In ^18^F-ML-10 imaging, the values of tumor/muscle (T/M) between CNE1 and CNE2 mice were statistically different at both 24 h and 48 h after irradiation. Besides, ΔT/M_1-0_ and ΔT/M_2-0_ of CNE2 mice were higher than those of CNE1 mice, demonstrating obvious discrepancy. Furthermore, we observed obvious changes of radioactive distribution in CNE2 group. On the contrary, T/M of ^18^F-FDG in irradiation group revealed no obvious change in both CNE1 and CNE2 groups. In conclusion, ^18^F-ML-10 animal PET/CT showed its potential to predict radiosensitivity in NPC.

## INTRODUCTION

Nasopharyngeal carcinoma (NPC) is an umbrella term for a group of malignant epithelium-originated tumors with different etiopathogenesis and a broad range of histopathological appearances [1]. It is distinct from other squamous cell carcinomas of the head and neck and shows high incidence rate in Southeastern Asia, including Malaysia, Indonesia, Singapore and China [2]. Radiotherapy or comprehensive chemotherapy administered before radiotherapy has been performed routinely for NPC therapy at the present due to its specific biologic behavior and anatomic characteristics. Although NPC is vulnerable to the radiation [3]; in addition, the advance of Intensive-Modulated Radiation Therapy (IMRT) and induction chemotherapy have improved tumor control and survival in NPC patients [4-6]; local residual disease still occurs in approximately 7 %–13 % after primary treatment for NPC; and tumor recurrence leads to a poor prognosis [7-9]. It's remarkable to predict a particular tumor's radiosensitivity before or during early stages of treatment so as to optimize treatment strategy and decrease tumor progression. It has been reported that the radiosensitivity is associated with apoptosis, autophagy, hypoxia, angiogenesis and DNA damage [10-12]. Therefore, the patients may benefit if we can observe the changes of tumor apoptosis dynamically and make individualized treatment decision according to the results.

Positron emission tomography (PET) molecular imaging, as a noninvasive modality, could characterize and monitor the biological processes at the cellular and molecular levels. Recently, a small molecular PET probe, 2-(5-[^18^F]fluoropentyl)-2-methyl malonic acid (^18^F-ML-10), which selectively targets cells undergoing apoptosis and is not taken up by necrotic cells, has been identified as a potent PET radiotracer for imaging the apoptosis [13]. In this study, we hypothesized that ^18^F-ML-10 PET/CT apoptosis imaging could predict the radiosensitivity *in vivo*. Hence, we established animal models with different levels of radiosensitivity by using CNE1 and CNE2 cell lines in order to verify our hypothesis. Besides, ^18^F-fluoro-deoxy-glucose (^18^F-FDG) PET/CT imaging was performed as routine control group.

## RESULTS

### Irradiation reduced tumor volume

**Figure 1:**
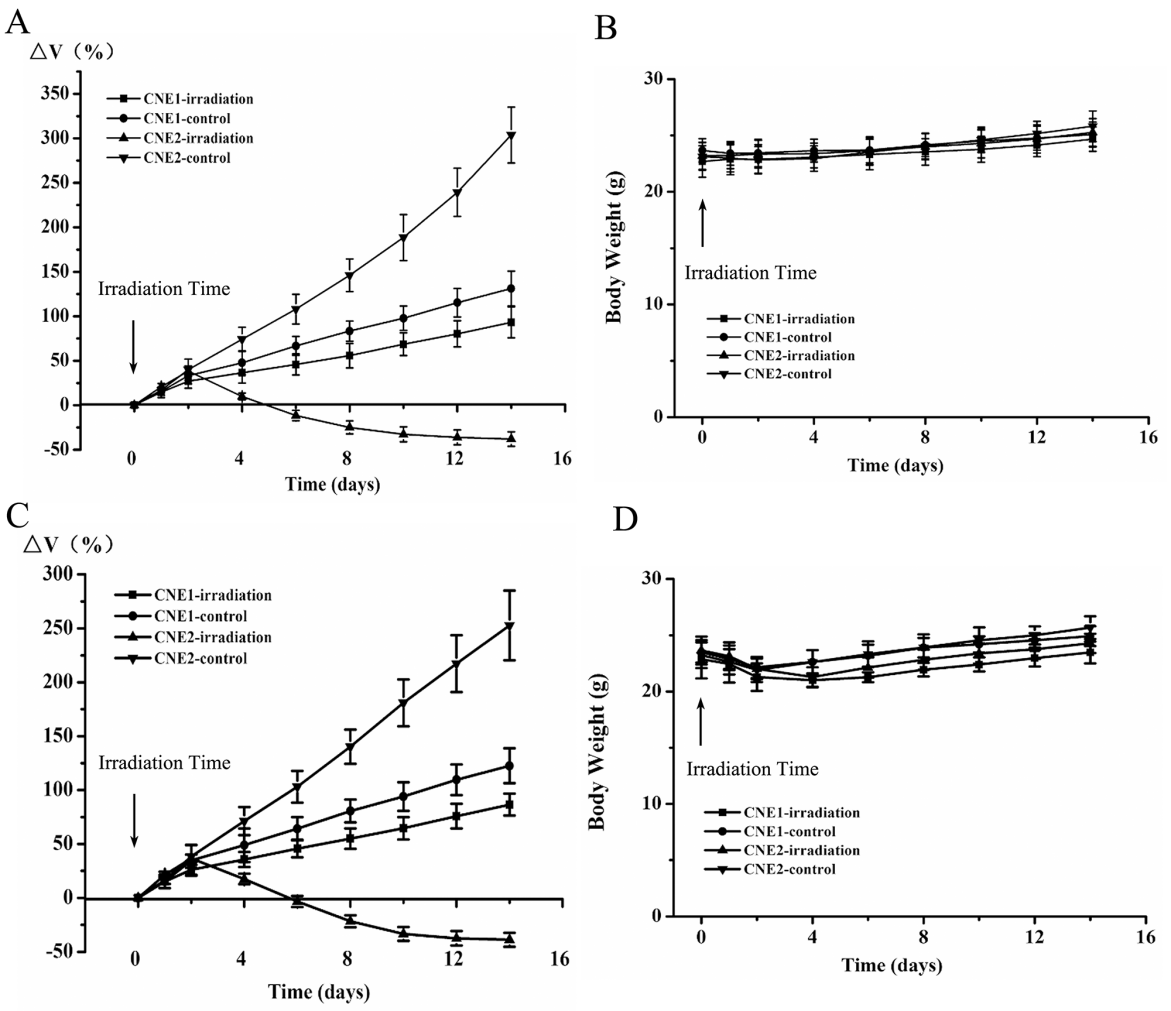
The volume changes and body weight of CNE1 and CNE2 mice in irradiation and control groups at different times ^18^F-ML-10 PET/CT group **A, B.** and ^18^F-FDG PET/CT group **C, D.**

The volume changes of CNE1 and CNE2 mice were shown in Figure 1A, 1C. As expected, CNE2 tumors decreased significantly while CNE1 tumors increased gradually after irradiation. Simultaneously, a time-related increase in tumor volume was observed in the two control groups. There was significant difference of ΔV_x_ between CNE1 and CNE2 tumors from 2 days after irradiation (*P*<0.05).

As shown in Figure 1B, 1D, no significant loss of body weights was observed during this 2-week study (*P*>0.05). It indicated that 15 Gy irradiation had no obvious toxic side effects at the prescribed protocol.

### Irradiation induced tumor cell apoptosis

We used ^18^F-ML-10 animal-PET/CT to assess tumor cell apoptosis induced by irradiation. As shown in Table 1, we found no difference of T/M_0_ between irradiation and control group both in CNE1 and CNE2 tumors (*P*>0.05), suggesting that they were homogeneity.

**Table 1:** The Value of ^18^F-ML-10 T/M in CNE1 and CNE2 Mice

T/M	CNE1	CNE2	P*
Irradiation group	(*n* = 11)	(*n* = 11)	
T/M_0_	4.77 ± 0.71	5.31±0.61	0.069
T/M_1_	4.96 ± 0.58	7.59 ± 0.84	< 0.001
*P*^#^	0.272	< 0.001	
T/M_2_	5.06 ± 0.78	9.89 ± 0.66	< 0.001
*P*^#^	0.114	< 0.001	
Control group	(*n* = 5)	(*n* = 5)	
T/M_0_	4.77 ± 0.58	5.30 ± 0.50	0.16
T/M_1_	4.80 ± 0.52	5.55 ± 0.53	0.054
*P*^#^	0.68	0.153	
T/M_2_	4.84 ± 0.58	5.74 ± 0.54	0.034
*P*^#^	0.783	0.075	
*P*^+^	0.658	0.583	

P* values are for the difference of T/M_n_ between CNE1 and CNE2.

P# values are for the difference between T/M_n_ and T/M_0_.

P+ values are for the difference of T/M_0_ between irradiation and control group.

**Figure 2:**
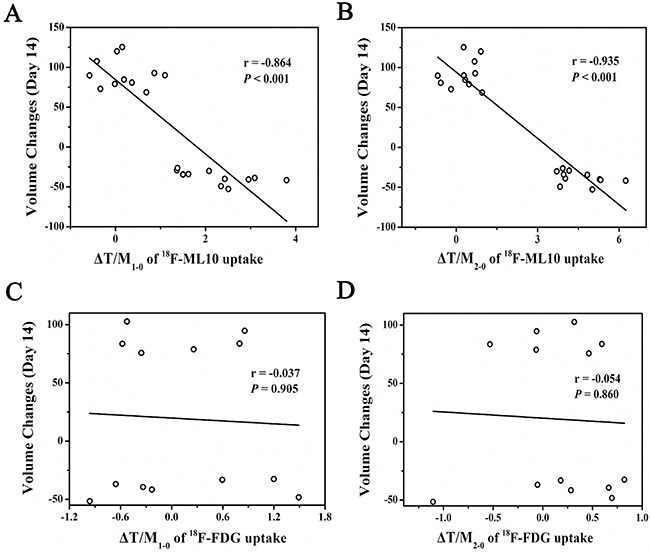
The scatter plot showed ΔT/M_1-0_ A. and ΔT/M2-0 B. of ^18^F-ML-10 was negatively correlated with ΔV_14_, respectively; ΔT/M_1-0_ C. and ΔT/M_2-0_ D. of ^18^F-FDG was uncorrelated with ΔV_14_, respectively

In irradiation group, the value of tumor-to-muscle ratio (T/M) in CNE1 and CNE2 mice has shown statistically difference at both 24 h (4.96 ± 0.58 versus 7.59 ± 0.84, *P* < 0.001) and 48 h (5.06 ± 0.78 versus 9.89 ± 0.66, *P* < 0.001) after irradiation. T/M of CNE1 mice had shown gently increased tendency, but no statistical difference was figured out within both 24 h and 48 h (*P*>0.05). However, in CNE2 tumors, significant differences of T/M were observed both at 24 h (T/M_0_ = 5.31±0.61 versus T/M_1_ = 7.59 ± 0.84, *P*<0.001) and 48 h (T/M_0_ = 5.31±0.61 versus T/M_2_ = 9.89 ± 0.66, *P*<0.001) after irradiation compared with the baseline. Consequently, ΔT/M_1-0_ and ΔT/M_2-0_ of CNE2 mice were higher than that of CNE1 mice, displaying obvious discrepancy (Table 2). In irradiation group, ΔT/M_1-0_ and ΔT/M_2-0_ showed negative correlation with the therapeutic effect (ΔV_14_) respectively(*r* = −0.864, *P*<0.001; *r* = −0.935, *P*<0.001) (Figure 2A and 2B).

**Table 2:** The Value of ^18^F-ML-10 ΔT/M in CNE1 and CNE2 irradiation Mice

	CNE1 (*n* = 11)	CNE2 (*n* = 11)	P*
ΔT/M_1-0_	0.20 ± 0.51	2.28 ± 0.79	<0.001
ΔT/M_2-0_	0.29 ± 0.55	4.57 ± 0.82	<0.001
*P*^#^	0.592	<0.001	

P* values are for the difference of ΔT/M_n-0_ between CNE1 and CNE2.

P# values are for the difference between ΔT/M_1-0_ and ΔT/M_2-0_.

In control group, T/M of both CNE1 and CNE2 mice displayed its slight uptrend, but no statistical difference was detected (*P*>0.05).

### Irradiation changed the distribution of tumor apoptosis

**Figure 3:**
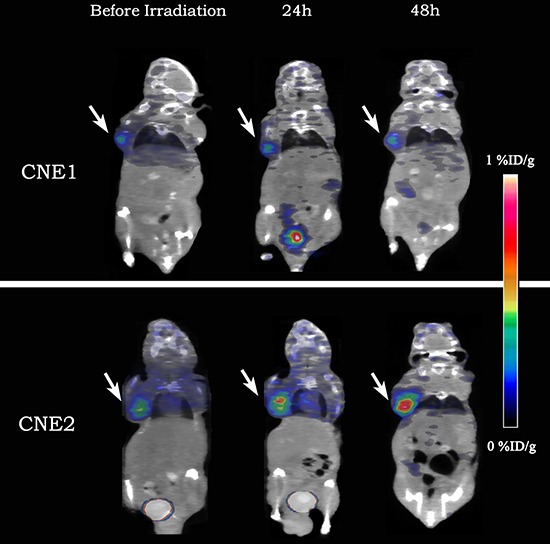
Representative decay-corrected whole-body coronal ^18^F-ML-10 animal-PET/CT images of CNE1 and CNE2 groups before and 24 h, 48 h after irradiation The uptake of ^18^F-ML-10 was stable in CNE1 tumor-bearing nude mouse (the upper) while increased greatly in CNE2 one (the lower).

Figure 3 showed representative coronal animal-PET/CT infusion images of CNE1 and CNE2 tumor-bearing nude mice. Before irradiation, CNE1 and CNE2 tumors exhibited a region of relatively high ^18^F-ML-10 uptake in the tumor. At 24 h and 48 h after irradiation, the peak uptake of radioactive tracer remained in the same region in CNE1 tumor. However, in CNE2 tumors, the peak uptake of ^18^F-ML-10 shifted to another region at 24 h after irradiation, and then shifted once again with an extended range of radioactive distribution at 48 h after irradiation. It was found that 8 of 11 CNE1 mice have kept the same region of peak uptake. 4 of 10 CNE2 mice extended the range of radioactive distribution, and 5 CNE2 mice revealed both change of peak-shift and range-extend. *P* value of Fisher's Exact Test was 0.008 (Table 3).

**Table 3:** Changes of Radioactive Distribution after Irradiation in ^18^F-ML-10 imaging

Change of radioactive distribution	Changed			Unchanged	*P*
	Peak-shift	Range-extend	Both		
CNE1 (*n* = 11)	3	/	/	8	0.0008
CNE2 (*n* = 11)	1	4	5	1	

P values is the two sided probability of the Fisher's Exact Test.

### Irradiation does not affect tumor glucose metabolism

**Figure 4:**
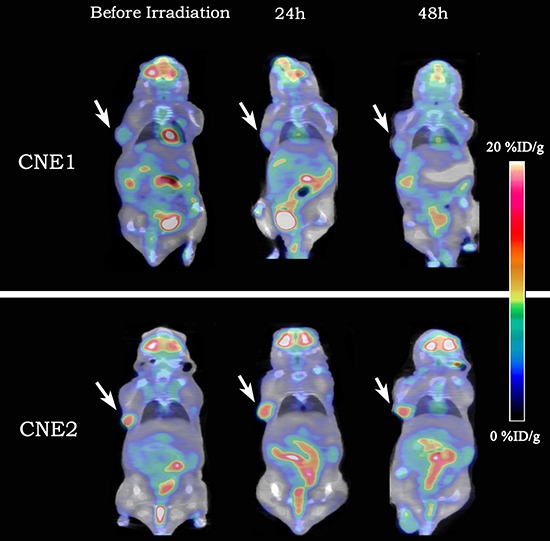
Representative decay-corrected whole-body coronal ^18^F-FDG animal-PET/CT images of CNE1 and CNE2 groups before and 24 h, 48 h after irradiation The uptake of ^18^F-FDG showed on obvious change in both CNE1 tumor-bearing nude mouse (the upper) and CNE2 one (the lower).

^18^F-FDG animal-PET/CT imaging is routinely applied to quantitatively measure the glucose metabolism of tumor induced by irradiation. Representative coronal animal-PET/CT infusion images of CNE1 and CNE2 tumor-bearing nude mice were shown in Figure 4. There was no significant difference of T/M both at 24 h and 48 h after irradiation compared with the baseline in CNE1 and CNE2 tumors (Table 4). However, T/M in CNE2 tumors was higher than that in CNE1 tumors, showing CNE2 cells were more poorly differentiated. Meanwhile, T/M of both CNE1 and CNE2 mice had shown gently increased tendency. Consequently, ΔT/M_1-0_ and ΔT/M_2-0_ in CNE1 and CNE2 mice were also no obvious difference (Table 5). In irradiation group, ΔT/M_1-0_ and ΔT/M_2-0_ were uncorrelated with the therapeutic effect (ΔV_14_) (Figure 2C and 2D).

**Table 4:** The Value of ^18^F-FDG T/M in CNE1 and CNE2 Mice

T/M of ^18^F-FDG	CNE1	CNE2	P*
Irradiation group	(*n* = 6)	(*n* = 7)	
T/M_0_	5.15 ± 0.51	8.94 ± 0.97	< 0.001
T/M_1_	5.23 ± 0.96	9.10 ± 1.02	< 0.001
*P*^#^	0.789	0.676	
T/M_2_	5.27 ± 0.73	9.16 ± 0.86	< 0.001
*P*^#^	0.516	0.427	
Control group	(*n* = 4)	(*n* = 5)	
T/M_0_	5.18 ± 0.80	8.97 ± 0.82	< 0.001
T/M_1_	5.20 ± 0.38	9.08 ± 0.81	< 0.001
*P*^#^	0.951	0.528	
T/M_2_	5.16 ± 0.41	9.03 ± 0.74	< 0.001
*P*^#^	0.962	0.722	
*P*^+^	0.847	0.886	

P* values are for the difference of T/M_n_ between CNE1 and CNE2.

P# values are for the difference between T/M_n_ and T/M_0_.

P+ values are for the difference of T/M_0_ between irradiation and control group.

**Table 5:** The Value of ^18^F-FDG ΔT/M in CNE1 and CNE2 irradiation Mice

	CNE1 (*n* = 6)	CNE2 (*n* = 7)	P*
ΔT/M_1-0_	0.075 ± 0.65	0.16 ± 0.95	0.862
ΔT/M_2-0_	0.12 ± 0.42	0.21 ± 0.66	0.772
*P*^#^	0.882	0.83	

P* values are for the difference of ΔT/M_n-0_ between CNE1 and CNE2.

P# values are for the difference between ΔT/M_1-0_ and ΔT/M_2-0_.

### The value of ^18^F-ML-10 T/M in predicting radiosensitivity

According to the volume changes, we could regard CNE2 group was much more sensitive to radiation than CNE1 group. As has been shown in above, ΔT/M_1-0_ and ΔT/M_2-0_ of ^18^F-ML-10 were different in these two groups. By means of a receiver operating characteristic curve, the optimal cut value of ΔT/M_1-0_ and ΔT/M_2-0_ to predict responder were −1.57 and −1.68, respectively (both sensitivity and specificity=100.0%).

### ^18^F-ML-10 accumulation correlated well with TUNEL

**Figure 5:**
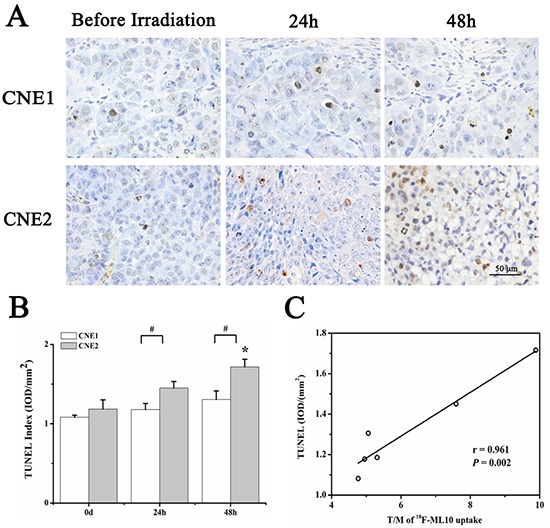
TUNEL analysis of CNE1 and CNE2 tumor sections before and 24 h, 48 h after irradiation Representative captures of TUNEL staining **A.** TUNEL index in both CNE1 and CNE2 groups at different time points **B.** Correlation analysis between T/M of ^18^F-ML10 uptakes and apoptosis index **C.** *P<0.05, within CNE2 group, compared to day 0. # *P*<0.05, between CNE1 and CNE2 groups.

To confirm the presence of apoptotic cells in tumor, TUNEL staining was performed on tumor sections from irradiation group. Representative captures of TUNEL staining were shown in Figure 5A. Consistent with ^18^F-ML-10 uptakes, TUNEL index emerged escalating trend in both CNE1 and CNE2 groups (Figure 5B). Correlation analysis between T/M of ^18^F-ML-10 uptakes and apoptosis index revealed a correlation of 0.961(*P* = 0.002) (Figure 5C).

### ^18^F-FDG uptake correlated well with Glut-1

**Figure 6:**
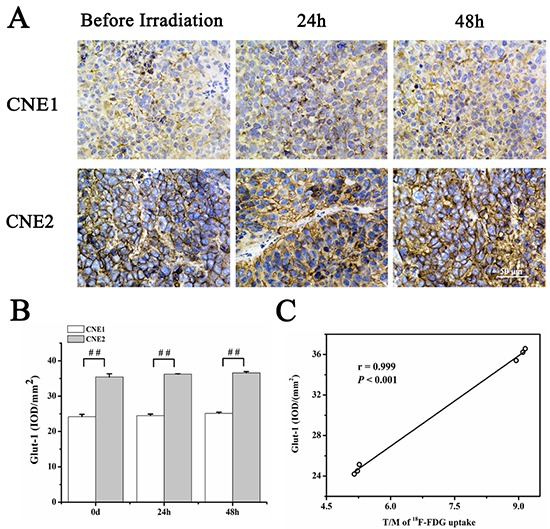
Glut-1 analysis of CNE1 and CNE2 tumor sections before and 24 h, 48 h after irradiation Representative captures of Glut-1 staining **A.** Glut-1 intensity in both CNE1 and CNE2 groups at different time points **B.** Correlation analysis between T/M of ^18^F-FDG uptakes and Glut-1 expression **C.** # *P*<0.01, between CNE1 and CNE2 groups.

Glut-1 staining was also performed to verify tumor glucose metabolism in irradiation group. Figure 6A illustrated representative captures of Glut-1 staining. As was expected, Glut-1 intensity had no obvious change in both CNE1 and CNE2 groups, yet higher in the former group (Figure 6B). T/M of ^18^F-FDG uptakes and Glut-1 intensity revealed a positive correlation of 0.999 (*P* < 0.001) (Figure 6C).

## DISCUSSION

Since radiotherapy is one of the primary treatment means for nasopharyngeal carcinoma and disease response varies among patients, it's crucial to predict a particular tumor's radiosensitivity before or during early stages of treatment in order to guide individual therapy management. At the present, imaging technologies, especially some noninvasive molecular modalities, such as PET/CT, play more and more important roles in modern cancer care [14, 15]. ^18^F-FDG PET/CT, which has been routinely used in oncology, can provide functional or metabolic characteristics of malignancies while conventional imaging modalities predominantly detect anatomical or morphologic features [16, 17]. However, ^18^F-FDG does not give a direct measure of cell growth and its false positive pitfall in inflammation would limit the application in some cases [18]. Just as this study,^18^F-FDG could not evaluate early response of irradiation both in CNE1 and CNE2 tumors. Therefore, other radiolabelled molecular probes, which could detect radiosensitivity more efficiently, should be investigated.

In our study, we used CNE1 and CNE2 nasopharyngeal carcinoma xenografts, which on behalf of different levels of differentiation and radiosensitivity, to verify the ability of predicting early response to irradiation via ^18^F-ML-10 PET/CT apoptosis imaging. We found that ^18^F-ML-10 longitudinal animal-PET/CT imaging successfully predicted radiosensitivity of CNE1 and CNE2 xenografts and observed the change of tumor apoptosis distribution early after irradiation at 24 h and 48 h after irradiation. Two days after irradiation, CNE1 and CNE2 tumors began to shrink, but poorly differentiated CNE2 tumors revealed a sharper and greater decrease compared to highly differentiated CNE1 tumors. As previous study has shown that the poorly differentiated NPC would be more radiosensitive than highly ones [19], we could regard CNE2 group as irradiation responder while CNE1 group as non-responder. Merely 24 h after irradiation, T/M_1_ value of ^18^F-ML-10 uptake in CNE2 tumors, namely irradiation responder, was significantly higher than that in CNE1 tumors. We hypothesized higher T/M_n_ or ΔT/M in apoptosis imaging might lead to better response to radiotherapy, and our study verified the results. The tumor apoptosis induced by radiation was supported by increased TUNEL staining index. In our previous study, ^18^F-FLT animal-PET/CT imaging illustrated that proliferation of CNE2 tumors decreased after radiotherapy, which corroborated each other with current study [20].

^18^F-ML-10 is derived from the Aposense family of biomarkers for apoptosis, with a small molecule weight of 206 [21]. As PET tracers, small-molecular-weight compounds may be superior to large protein-based probes such as annexin-V. The advantages include better biodistribution and less immunologic responses [22–24]. In the first human study, ^18^F-ML-10 showed a quick excretion from blood through the kidneys and rapid clearance from nontarget organs, resulting to a high and stable organ-to-blood ratio from 30 min after probe administration [22–24]). Besides the increase of T/M value, ^18^F-ML-10 PET scan showed a different tracer distribution at 24 h and 48 h after irradiation compared to baseline in radiosensitive tumors. In a newly diagnosed glioblastoma multiforme patient treated with whole-brain radiation therapy, ^18^F-ML-10 uptake reduced at the site of greatest baseline uptake, but increased uptake around the periphery of the tumor [25]. This changing pattern of ^18^F-ML-10 uptake was similar with the peak-uptake-shift in our study, suggesting radiation-induced tumor cellular apoptosis may be not stationary. This phenomenon may prompt a new irradiation mode: adjusting radiotherapy plan in real time according to ^18^F-ML-10 PET scan. Furthermore, treatment doses should be increased for patients with poor radiosensitivity and for regions with low apoptosis, whereas doses should be decreased for patients with high radiosensitivity and for regions with high apoptosis. Thus, complications could be reduced without cutting down curative effects.

Our study was the first to report the potential value of ^18^F-ML-10 PET/CT in predicting radiosensitivity of NPC. Moreover, we elaborated the change of apoptosis distribution after radiotherapy. If further human clinical trial also showed the satisfied results, we hope ^18^F-ML-10 PET/CT could be used in every NPC patients’ radiotherapy treatment planning.

## MATERIALS AND METHODS

### Cells culture

Highly differentiated human nasopharyngeal squamous cell carcinoma cell line CNE1 and poorly differentiated nasopharyngeal squamous cell carcinoma cell line CNE 2 were kindly given by Professor Jianji Pan (Department of Radiation Oncology, Fujian Tumor Hospital, Provincial Clinical College of Fujian Medical University, Fuzhou, Fujian, China). The cells were cultured in RPMI-1640 medium enriched with 10% fetal calf serum and mixture antibodies of 100 units/mL penicillin and 0.1 mg/mL streptomycin (all from Gibco BRL, Life Technologies, Rockville, MD, USA) at 37°C in a humidified atmosphere with 5 % CO_2_, and kept in log phase by routine passage every 2-3 days. The cells were subsequently collected by trypsinization with 0.25 % trypsin/EDTA.

### Animal models

The experiment was approved by our institution (Institutional animal care and use committee number, 20150392A103). All procedures involving animals were performed in accordance with institutional guidelines (Guide for the Care and Use of Laboratory Animals of Fudan University, Shanghai, China). A total number of 74 male athymic Balb/c nude mice (5 weeks) were obtained from Department of Laboratory Animal Science, Fudan University and allowed to acclimatize for one week in the animal facility before any intervention was initiated. Mice were randomly divided into two groups, and then injected with 0.1 mL cell suspension (1×10^7^ cells in 1 mL RPMI-1640) of either CNE1 or CNE2 in armpit of right forelegs. Animals were housed in ventilated caging conditions under a 12-h dark/light cycle at constant humidity and temperature. They were allowed free access to sterile water and standard laboratory chow.

Among them, 56 of 74 mice were divided into imaging groups. In addition, 18 of 74 nude mice were used for immunohistochemistry staining.

### Irradiation

When the tumors reached nearly 8 mm in diameter (one week after inoculation of CNE2 and two weeks after inoculation of CNE1), they were anesthetized with 0.4 mL 1 % pentobarbital sodium via intraperitoneal injection. Xenografts were covered with gauze coated in 10 mm of petroleum jelly and received a single fraction of 15 Gy by an animal specific accelerator (SARRP, Gulmay Medical Inc, Suwanee, Georgia, USA).

### Synthesis of ^18^F-ML-10

^18^F was produced in-house using a cyclotron [Eclipse ST (40 μA × 11 MeV); Siemens, Knoxville, Tennessee, USA]. Radiolabeling of ML-10 with ^18^F was synthesized at our center according to the method described by Wang *et al* [26]. The radiochemical purity of ^18^F-ML-10 was more than 97%.

### Animal-PET/CT imaging

Animal-PET/CT scans and image analyses were performed 1 hour after injection of radiolabelled tracer (via tail vein with 5.55 MBq ^18^F-FDG or ^18^F-ML-10 in 0.2 mL saline) using an Inveon Animal-PET/CT (Siemens Preclinical Solution, Knoxville, TN) before and 24 h, 48 h after irradiation. 32 mice were scanned with ^18^F-ML-10, and 24 mice were performed with ^18^F-FDG. Animals were maintained under 2 % isoflurane anesthesia during scanning period. Besides, mice in the ^18^F-FDG group were fasted 4h before probe injection, maintained under isoflurane anesthesia and kept warm during injection, waiting phase, and scanning periods.

The mice were placed in prone position on the bed of the scanner and two bed positions were acquired (five-minute CT scanning followed by ten-minute PET scanning). The animal-PET and animal-CT images were generated separately and then fused using Inveon Research Workplace (Siemens Preclinical Solution, Knoxville, TN). Three-dimensional ordered-subset expectation maximization (OSEM3D)/maximum algorithm was used for image reconstruction. The region of interest (ROI) was manually drawn covering the whole tumor on the fused images for further analysis. Additionally, a sphere region of interest was drawn on the muscle of the opposite foreleg of the mouse on the fused images. The highest uptake point of entire tumor was included in ROI and no necrosis area was allowed. The max of percentage-injected dose per gram (%ID/g_max_) of the tumor and muscle in the ROIs were recorded. The T/M was calculated by dividing %ID/g_max_ of the tumor by that of the muscle. T/M before and 24 h, 48 h after irradiation were defined as T/M_0_ and T/M_1_, T/M_2_ respectively; and its changes after irradiation were defined as ΔT/M_1-0_ and ΔT/M_2-0_, which meant (T/M_n_-T/M_0_)/T/M_0_.

### Immunohistochemistry

Before and 24 h, 48 h after irradiation, three mice in each CNE1 and CNE2 irradiation groups were sacrificed and tumor samples were paraffin embedded to perform TUNEL and Glut-1 staining. Image-Pro Plus (6.0) software was used to assess TUNEL positive number of nuclei and human Glut-1 intensity. TUNEL index and Glut-1 intensity were calculated by measuring the integrated optical density (IOD) of images that were of equivalent area (mm^2^). For each tumor section, 6 random high-powered fields (200×) were analyzed.

### Xenograft volume and body weight

Caliper (model 530-312; range 0-150 mm; Mitutoyo, Kawasaki, Kanagawa, Japan) measurements of perpendicular axes of the tumor were performed to follow up tumor growth every the other day during the study. Mice body weights were recorded on the same day. The formula for the volumes of the xenografts were expressed as V_x_ (V_x_=*ab*^2^/2), where V_x_ is the volume of the xenograft in the *x* day after irradiation; *a* is the long diameter of the xenograft; and *b* is the short diameter of the xenograft. The change of the xenograft volume within *x* days after irradiation was defined as ΔV_x_ and ΔV_x_ = (V_x_-V_0_)/V_0_, where V_0_ is the volume of xenograft just before irradiation.

### Statistical analysis

Data was expressed as mean ± SD. A two-tailed one sample Kolomogorov-Simirnov test was utilized to examine the normality of quantitative data. The difference between CNE1 and CNE2 was tested by independent *t* tests. We added a correction to compensate for unequal variance in cases where variance between groups was unequal. For comparison of the differences inside one group, we instead used the paired *t* test. Pearson correlation *r* was performed to calculate the correlations between animal-PET/CT images and immunohistochemistry. Moreover, we used receiver operating characteristic (ROC) curves to acquire an optimal cut value to differentiate irradiation responder and non-responder. Youden's index (Youden Index=specificity+sensitivity-1) is often used in conjunction with ROC analysis, and the maximum value of the index is used as a criterion for selecting the optimum cut-off point. Data was analyzed by SPSS 20.0 software packages (IBM Corporation, Armonk, NY). All analyses were two-sided. A *P* value less than 0.05 was considered statistically significant.

In ^18^F-FDG PET/CT group, two mice from the CNE1 group expired due to anesthesia accidents during imaging. Therefore, the rest 54 mice were included for further analysis (^18^F-ML-10: 11 mice/CNE1 or CNE2 irradiation, 5 mice/CNE1 or CNE2 control; ^18^F-FDG: 6 mice/CNE1 irradiation, 7 mice/CNE2 irradiation, 4 mice/CNE1 control and 5 mice/CNE2 control).
